# Pharmacist-led DE-eSCALation of opioids post-surgical dischargE (DESCALE) – A multi-centre, non-randomised, feasibility study protocol

**DOI:** 10.3310/nihropenres.13716.1

**Published:** 2024-08-22

**Authors:** Emma L Veale, Johanna Theron, Melanie Rees-Roberts, Julie H Hedayioglu, Ellie Santer, Sabina Hulbert, Vanessa J Short

**Affiliations:** 1 Medway School of Pharmacy, University of Kent and University of Greenwich, Chatham Maritime, ME4 4TB, UK; 2 Community Chronic Pain Team, Kent Community Health NHS Foundation Trust, Margate, CT9 1LB, UK; 3 Centre for Health Services Studies, University of Kent, Canterbury, England, CT2 7NF, UK; 4 Research & Development, Kent Community Health NHS Foundation Trust, Ashford, England, TN25 4AZ, UK

**Keywords:** Opioids, deprescribing, surgery, dependence, clinical pharmacists, Medicines Use Review, primary care, secondary care

## Abstract

**Background:**

Opioids are frequently prescribed for short-term acute pain following surgery. Used appropriately, opioids deliver extremely favourable pain relief. Used longer than 90-days, however, can result in health complications, including unintentional overdose and addiction. Globally, >40 million people are dependent on opioids and annually >100,000 die from opioid misuse. With >4.7 million surgical procedures occurring annually in the United Kingdom it is imperative that opioid-use is managed upon discharge. A declining General Practitioner (GP) workforce and increased patient numbers, however, means gaps in healthcare during transfer of care. Here we report a mixed-methods protocol to understand the feasibility, and acceptability of a clinical pharmacist (CP)-led early opioid deprescribing intervention for discharged surgical patients.

**Methods:**

DESCALE is a multicentre, non-randomised, pragmatic feasibility study. Participants aged ≥18 years who have undergone a surgical procedure at a single NHS trust in Southeast England and discharged with opioids and without a history of long-term opioid use, cancer diagnosis or study contraindications will be offered a Medicines Use Review (MUR) within 7-10 days of discharge. The MUR will be delivered by CPs at participating GP practices. Feasibility outcomes will focus on recruitment, fidelity of CPs to deliver the MUR, and barriers within primary care that affect delivery of the intervention, with a maximum sample size of 100. Clinical outcomes will focus on the number of participants that reduce or stop opioid use within 91 days. Prescribing, medical, surgical, and demographic data for individual participants will be collected and analysed to inform future trial design. Qualitative interviews with participants and associated healthcare professionals will explore acceptability and implementation of the intervention.

**Conclusion:**

Data collected with respect to opioid use post-surgery, feasibility and acceptability of the intervention, patient experience and outcome data will inform the design of future research and larger clinical trials.

## Plain Language Summary

Opioids are very strong pain killers often given to help with severe pain, such as after having surgery. Used for a short time these drugs are very good at taking away pain. When used longer than the recommended 2-4 weeks, opioids can cause more pain and become addictive or even, the risk of early death. Despite the high side effects from using opioids, the numbers of people given these drugs has risen sharply. Whilst it’s important that surgical pain is treated correctly, it is equally important that opioids are not continued for longer than is needed and is safe. Almost 5-million surgeries occur every year in the United Kingdom (UK), yet there are no national guidelines for healthcare professionals giving out opioids after surgery. This risks these sorts of drugs being used for too long by patients. In the UK, East Kent has been highlighted as a region with above average use of these drugs by patients and use for longer than is needed and safe.

This study is funded by the National Institute for Health Research, approved by the North-West Greater Manchester Central Research Ethics Committee, and designed with public and patient involvement groups. Learning from this study will help us understand if it is feasible for pharmacists based in GP surgeries to speak with, and support patients given opioid drugs after surgery, and ensure these drugs are used for only the amount of time needed. It will also show us what improvements need to be made before conducting a larger study.

We are working with all three hospitals within East Kent University Foundation Trust and six participating GP practices to recruit 100 patients to take part. Results will be shared in NHS websites/newsletters, in community magazines and in academic journals.

## Introduction

### The opioid problem

Opioids are very strong painkillers that are frequently prescribed to treat short-term acute pain, such as after having a surgical procedure. Used for acute or end of life pain, these drugs deliver favourable analgesic pain-relief. Opioids can however cause serious health risks and side effects such as more pain, medication dependence and in some cases unintentional overdose or premature death from misuse if used for a long period of time (>90-days) ^1, 2^. In the United States (US), a White House commissioned report in 2017 into opioid misuse, found that it was responsible for the death of 64,000 people annually and the leading cause of death related to unintentional injury ^3, 4^. In the United Kingdom (UK), during the period 2020-2021, almost half (49.6%) of all drug-related deaths involved an opioid ^5^. Despite the high potential for serious adverse events when using opioids, the global numbers of people using opioids, over the past two decades has risen dramatically, along with an associated increase in long-term dependency and mortality ^6– 10^. In 2017, it was estimated that 40.5 million people were classified as ‘dependent’ on opioids and 109,500 people had died from opioid overdose, worldwide ^10^. The first few days of opioid therapy have been shown to be crucial in determining the likelihood of long-term opioid use. Patients receiving opioids for longer than a week, were found to double their risk of using opioids for more than a year, which doubled again if opioids were used for longer than a month ^11, 12^. Whilst another study has shown that the cumulative opioid dose dispensed in the first month was also strongly related to the probability of long-term use, with long-acting drugs being worse, than short-acting drugs ^13^.

### Surgical opioid use

Opioids are routinely used pre-, intra-, and post-surgically for the treatment of acute pain and post-operatively for pain management and is a primary route to exposure to these types of medicines, often for the first time ^14, 15^. Evidence from the US and Canada has shown a clear link between post-surgical opioid prescribing for both minor and major surgery, and persistent long-term use in the community, beyond tissue healing. Inappropriate prescribing of opioids both peri- and post-operatively, accounts for 36.5% of all opioid prescribing in the US, with 3 – 10% of these patients transitioning to long-term use ^11, 14, 16, 17^. Patients undergoing surgery are almost four times more likely to be discharged with opioids than their non-surgical counterparts ^18^. This is often with more opioid medication than is actually required to treat post-surgical pain ^19– 22^. Perhaps more alarmingly, almost half (45%) of these patients are given opioids even though they would not have been taking any opioids at point of discharge ^23^. In the United States (US), a study showed that between 5.9 – 6.5% of all patients who underwent surgery (versus 0.4% of the non-surgical control) went on to become long-term opioid dependent, even if they had never taken opioids before ^15^.

It is becoming clear from the US, that postsurgical prescribing of opioids is a problematic, underexplored, new source of long-term opioid use ^17^. Compared to the US and Canada, less is known in the UK about peri-operative opioid prescribing in hospitals and any link to long-term use. Two recent studies in the UK have identified long-term opioid use (>1 year) to be associated with older age (>75 years), being socioeconomically disadvantaged, having a history of depression and/or alcoholism, prior gapapentinoid or psychotrophic use, and those initiated on >120 morphine milligram equivalents (MME) following major surgery ^6, 24^. There are over 5.6 million opioid prescriptions dispensed each year in the UK, corresponding to around 13% of the adult UK population ^25^ and over 4.7 million surgical procedures occurring each year ^26^ and nearly 8 million people on surgical waiting lists, with over 300,000 waiting more than 52 weeks for surgery ^27^. This, along with no current national guidelines on peri-operative opioid prescribing, a declining GP workforce and increasing patient numbers ^28^, policy makers and professional bodies are rightly concerned. In 2020, the Faculty of Pain Medicine and Royal College of Anaesthetists established a working party to set out ‘guiding principles’ in opioid management in the peri-operative period ^29^. In these guidelines, they acknowledge the critical role that surgery plays in the burgeoning opioid problem: “ *We have a duty to act to minimise the role that anaesthesia, surgery and primary care may have in contributing to the “opioid load” in the community in the UK. It is imperative that all healthcare professionals involved in surgery and perioperative care, work collaboratively to ensure robust opioid stewardship”* ^29, 30^. Whilst, the National Health Service (NHS) England Medicines Optimisation Executive Group have made reducing opioid use in chronic non-cancer pain a priority of the integrated care boards for 2023/2024 ^31^.

### Opioid problem in East Kent

Prescribing of high-dose opioids in East Kent has long been a growing concern within the NHS Kent and Medway Integrated Care Board and Integrated Care System (previously known as the Clinical Commissioning Group (CCG)). A recent report from Public Health England (PHE) highlighted East Kent Health and Care Partnership as being one of the top 30 highest opioid prescribers in England, with an opioid spend of £422,000/year above the national average and prescribing rates 3 – 4 times above the Kent and Medway CCG average ^25^. This has led to initiatives such as the East Kent High Dose Opioid Reduction project, a pharmacist-led intervention, established to tackle, high-dose long-term opioid users on >120 mg morphine milligram equivalent (MME) per day ^32^. Despite the success of such projects to reduce harmful levels of opioid use in the East Kent community, they do not allow us to understand the root source of the opioid problem from which these complex opioid users arise. Local prescribing and chronic pain clinical leads supporting this study are therefore keen to understand why these patterns of prescribing exist and what preventative opportunities might reduce prescribing and is where our study is based. Deprescribing opioids in a timely manner is important to minimise both the risk of any patient developing opioid tolerance and the possibility of any withdrawal symptoms or adverse events occurring. 

### Pharmacists and opioid management

Pharmacists are experts in medicines management and are trained to deliver high-risk Medicines Use Reviews (MURs), an activity that the public closely associate with the role of a pharmacist ^33– 35^. Indeed, in the UK, community pharmacists are contracted as part of the Community Pharmacy Contractual Framework ^36^ to deliver MURs to patients and by clinical pharmacists based in GP practices, as part of the additional role reimbursement scheme ^37^. However, for community pharmacists, access to a patient’s medical records and GP, can be a limiting factor ^38^. particularly when deprescribing medications such as opioids. Thus, clinical pharmacists employed in GP practices with access to patient hospital discharge lists, medical records and onsite support from GPs are ideally positioned to initiate early opioid deprescribing in post-surgical patients discharged back into the community.

### Aims and objectives

The primary aim of this study is to evaluate a multi-centre, pharmacist-led, early opioid deprescribing intervention. This is designed for patients discharged following a surgical procedure with an opioid prescription from hospitals within a single NHS Trust in an area of high opioid prescribing, East Kent. It will determine whether this designed intervention is able to support early deprescribing of opioids in this particular healthcare setting and with this particular patient population and to inform a much larger, clinical trial. A summary of the aims and objectives for this study are collated in Table 1.

**Table 1.** Summary of study aims and objectives.

Aims/questions	Objectives
**To test the feasibility of delivering an early opioid ** **deprescribing intervention in primary care, led by ** **trained clinical pharmacists.**	**Ascertain stakeholder acceptability of the intervention:** • Maintain detailed records of any barriers or enablers that may occur in response to setting up the intervention and whilst delivering the intervention • Deliver stakeholder interviews
	**Measure the capabilities of trained clinical pharmacists to deliver an ** **early opioid medication review by**: • Completed intervention delivery training • Observational competency checks by Clinical Lead • Completed medication review case reports • Number of participants successfully deescalated
	**Ascertain participant acceptability of the intervention by measuring**: • Number of participants that enrol in the study • Number of withdrawals from the study • Adherence to pharmacist medication recommendations • Responses to participant satisfaction questionnaire • Responses during participant interviews
	**Apply a micro-costing approach to estimate the intervention costs:** • opioid medication costs • staff costs • Health resource use (primary care services, community-based services, hospital inpatient and outpatient services)
**To measure successful discontinuation and/or ** **reduction in the use of opioids to within the ** **recommended 2–4-week period for opioid use ** **or by the expected period of tissue healing (<91 ** **days) whichever is sooner ^39^.**	**Measurement of** the mean difference in morphine milligram equivalent (MME) dose at baseline and at 90 days
**To understand post-surgical opioid prescribing ** **experiences including opioid use, long-term ** **opioid use, participant pain and quality of ** **life in the 3-month period post-surgery when ** **discharged with an opioid prescription**	• Collect medication use data at baseline and at all follow-up appointments • Compare opioid prescribing (type, dose, amount) at baseline for each surgical speciality and hospital • Measure pain scores at baseline and final visits • Measurement of risk factors in the participant population that have been previously linked to long-term opioid use ○ Baseline demographics; ○ Medical, prescription and surgical histories. • Response to Quality-Of-Life questionnaire (EQ5D-5L) complete at baseline and final appointment • Responses during participant interviews
**To ascertain the feasibility of delivering the ** **intervention as per the protocol to inform a ** **future study or adoption of the protocol**	**Measurements of:** • Time spent finding and recruiting participants • Number of possible surgical participants per week that could receive intervention • Number of pharmacists available to deliver the intervention • Number of surgical participants that accept an early MUR versus the number that are asked • Time taken from hospital discharge to receiving first MUR. • Average time taken for each appointment • Average time taken to successfully deescalate opioids in surgical patients • Number of participants that are successfully deprescribed opioids within 3 months • Number of patients that require additional appointments with the pharmacist • Number of participants that require additional support post-90 days • Responses during participant interviews

## Protocol

### Patient and Public Involvement

The patient and public involvement and engagement team (PPIE) consists of 3 National Institute for Health and Care Research (NIHR) Research Champions from Kent Community Health Foundation National Health Service (NHS) Trust, who have reviewed and modified the protocol and informed the content of the study, to ensure a patient-centred approach. In addition, each Research Champion has undergone qualitative research training, assisted in designing the interview schedule and are involved in delivering qualitative interviews to patient participants of the study. Two members of the public, with experience of receiving post-surgical opioids, participated in practice training MURs with the clinical pharmacists and two additional public contributors joined the research team, participating in monthly project meetings, assisting, and embedding their support in the project as it progresses. On completion of data collection, we will continue to work with the PPIE team to develop lay-friendly summaries of the findings for dissemination to the participants and public.

### Methods

**Figure 1.**
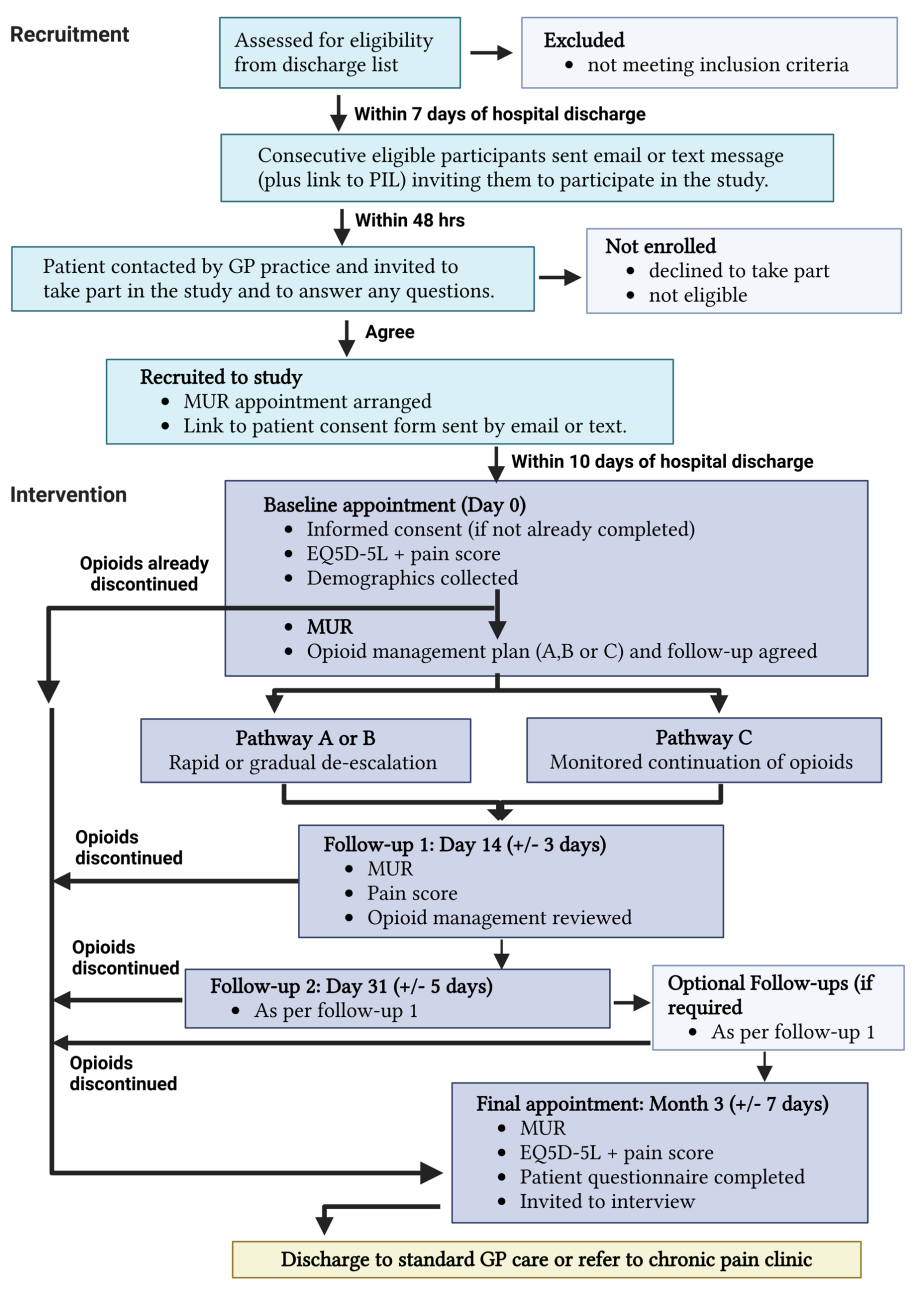
DESCALE Study Flow Chart. Abbreviations: PIL – patient information leaflet; hrs – hours; GP – general practitioner; MUR – medicines use review; EQ5D-5L – European Quality of Life 5 Dimension 5 Level survey. Image created with BioRender.com.

***Study design and setting.*** This is a prospective multi-centre, non-randomised, pragmatic feasibility sub-study, within the larger DESCALE study. The study involves clinical pharmacists based in GP practices in East Kent, initiating an early opioid MUR to support pain management and initiate opioid de-escalation, for surgical patients discharged from a hospital belonging to East Kent Hospitals University NHS Foundation Trust (EKHUFT). The study identifies potential participants from surgical discharge letters by practice staff, before an initial eligibility check, and consent to take part. A baseline MUR is conducted within 7 – 10 days of hospital discharge and continued monitoring and follow up MURs conducted as per the study design (see Study Flow Chart – Figure 1) until opioids have been discontinued or the participant is 3-months post-discharge ^39^. Recruitment of GP sites and participants, commenced in January 2024 and will continue until September 2024. We have currently recruited six primary care GP practices from East Kent to participate in the study.

### Trial registration

ClinicalTrials.gov: NCT06396663 (02/05/2024)

Accessed at: https://clinicaltrials.gov/study/NCT06396663

### Protocol version

Protocol Version 11, approval date 03.05.2024

### Eligibility criteria

***Inclusion criteria***

- Adults aged ≥18 years
- Undergone surgery in a hospital belonging to EKHUFT, who were discharged with opioid medication ≤120 mg MME/day and registered at a participating East Kent, GP practice.

***Exclusion criteria***

- Aged ≤18
- Unable to provide written informed consent
- On a dose of opioids >120 mg MME/day
- Taken opioids for >90 days prior to having surgery
- Taking opioids for malignant pain
- Follow-on surgery planned in the next 3 months
- Undergone a caesarean section
- Pregnant
- A history of using methadone or injecting opioids
- Considered vulnerable (e.g., severe dementia, severe co-existing or terminal medical condition).

### Recruitment

**Participants**: Participants identified as having had surgery and discharged with opioids (<120 mg) within the last 7 days via participating GP practice sites undertaking weekly database searches and/or by screening discharge letters will be contacted by either an email or text, inviting them to participate in the study and providing a link to the patient information leaflet (PIL). Invited participants will then be contacted by the practice using a consecutive sampling approach via telephone within 48 hours of the invite being sent, asking if they would like to take part in the study, answering any questions and confirming their eligibility. For patients that agree to participate, an MUR appointment (online, telephone or in person) will be arranged with the pharmacist and a link to an online consent form sent to the participate to complete.

**Sample size:** A sample size of 80 – 100 participants will be recruited. Statistically, sensitivity power analyses have confirmed that the proposed number is able to detect significant differences of the expected effect size and is in line with the recommended sample size of 70 to estimate key parameters, bench-marked from external pilot randomised controlled trials ^40^ and will allow for participant loss, to follow-up.

**GP practices:** By utilising networks locally an Expressions of Interest document was circulated to GP practices in Kent. Further to this, research active practices in East Kent and South Kent were approached directly and capacity and capability assessed. Moreover, the project’s Clinical Lead contacted clinical pharmacists who had interest in, or experience in research of opioids or pain, to invite them to express interest in recruiting via their practice(s).

**Clinical pharmacists:** GP practice-based clinical pharmacists with experience of delivering MURs and opioid deprescribing will be recruited from participating practices. The clinical pharmacist/s will receive detailed information about the study, undergo some training with the study Clinical Lead and will be asked to provide written informed consent.

### Consent process

Consent to participate in the study will be sought from all eligible patients that have undergone a surgical procedure and are discharged with an opioid prescription from the participating East Kent hospital sites and participating GP practices. Consent will only be sought after a full explanation and/or the PIL has been offered with time allowed for questions and proper consideration. Individuals willing to take part and for whom an MUR appointment has been made will be asked to complete a Participant Consent Form. A Consent form will be sent to those with digital capabilities prior to the MUR or completed together with the pharmacist at the first appointment. A typewritten signature or tick box declaration option, in accordance with the UK eIDAS Regulations (SI 2016/696) will be used for all digital consent forms. All consent forms will be checked and countersigned by the pharmacist prior to the commencement of the medication review. A copy of the consent form will be added to the participants medical records, and another retained in the study site folder, stored at the practice and a copy sent to the participant.

### Study interventions

Following recruitment and consent of eligible participants, a timely appointment (within ten days of hospital discharge) will be arranged with the pharmacist for an opioid MUR and biopsychosocial assessment. A detailed schedule of events can be found in Table 2.

**Table 2.** Participant timeline and schedule of events.

			Follow-up			
Study activity	Pre-intervention	Baseline (Day 0)	Day 14 (± 3 days)	Day 31 (± 5 days)	Month 3 (± 7 days)	Post- intervention
Eligibility check by Health Care Professional	**☑**	**☑**				
Informed consent	**☑**	**☑**				
Demographics		**☑**				
EQ5D-5L		**☑**	** ☑ ^a^**	** ☑ ^a^**	** ☑ ^a^**	
Pain Score		**☑**	**☑**	**☑**	**☑**	
MUR		** ☑ ^b^**	** ☑ ^b^**	** ☑ ^b^**	**☑**	
De-escalation pathway agreed		**☑**	**☑**	**☑**	**☑**	
Medication change form and prescription if required.		**☑**	**☑**	**☑**	**☑**	
Data Collection		**☑**	**☑**	**☑**	**☑**	
Participant experience questionnaire			**☑ ^a^**	**☑ ^a^**	**☑ ^a^**	
Participant interview invite			**☑ ^a^**	**☑ ^a^**	**☑ ^a^**	
Handover /discharge to standard care or pain clinic					**☑**	
Participant interview						**☑**

^**a**^Activities occurring at participants final appointment as determined by their individual de-escalation plans. **^b^** Optional follow-up appointments may be agreed depending on de-escalation plan and individual needs. Abbreviations: EQ5D-5L - European Quality of Life 5 Dimension 5 Level; MUR – medicines use review.

### Study MUR and biopsychosocial assessment protocol

During the intervention the clinical pharmacist will complete an online baseline data collection, case report form (CRF) where they will record:

➢ Surgical procedure undergone (hospital, ward, date, and length of stay)
➢ Opioid (drug name, dose per tablet (milligram (mg)), number of tablets/patches prescribed per day (24 hrs), total number of tablets prescribed)
➢ Opioid use prior to surgery (drug name, dose per tablet (mg), number of tablets/patches prescribed per day (8 am to 8 am), total number of tablets prescribed), length of time on opioids
➢ Calculate the combined morphine equivalent dose (MME, using Faculty of Pain Medicine guidelines) ^29^
➢ Record use of other analgesics including paracetamol, Non-Steroidal Anti-Inflammatory Drugs (NSAIDs) and other adjuvants – (drug name, dose per day)
➢ Determine if medically treated for depression or anxiety (if yes, drug name and dose) – taken from patient or retrieved from medical records, where applicable.
➢ Patient pain intensity in the last 24 hrs, measured using the Verbal Pain Rating Scale (No pain = 0; mild pain = 1; moderate pain = 2; severe pain = 3; very severe pain = 4)
➢ Record any coexisting morbidities and their effect on pain
➢ Record alcohol weekly consumption (1 – 7, 8 – 14, or >14 units*)
➢ Smoker (yes/no)

*One unit of alcohol is equivalent to 10 mL or 8 g of pure alcohol. E.g., 1 glass of wine (175 mL) = 2.1 units.

**Figure 2.**
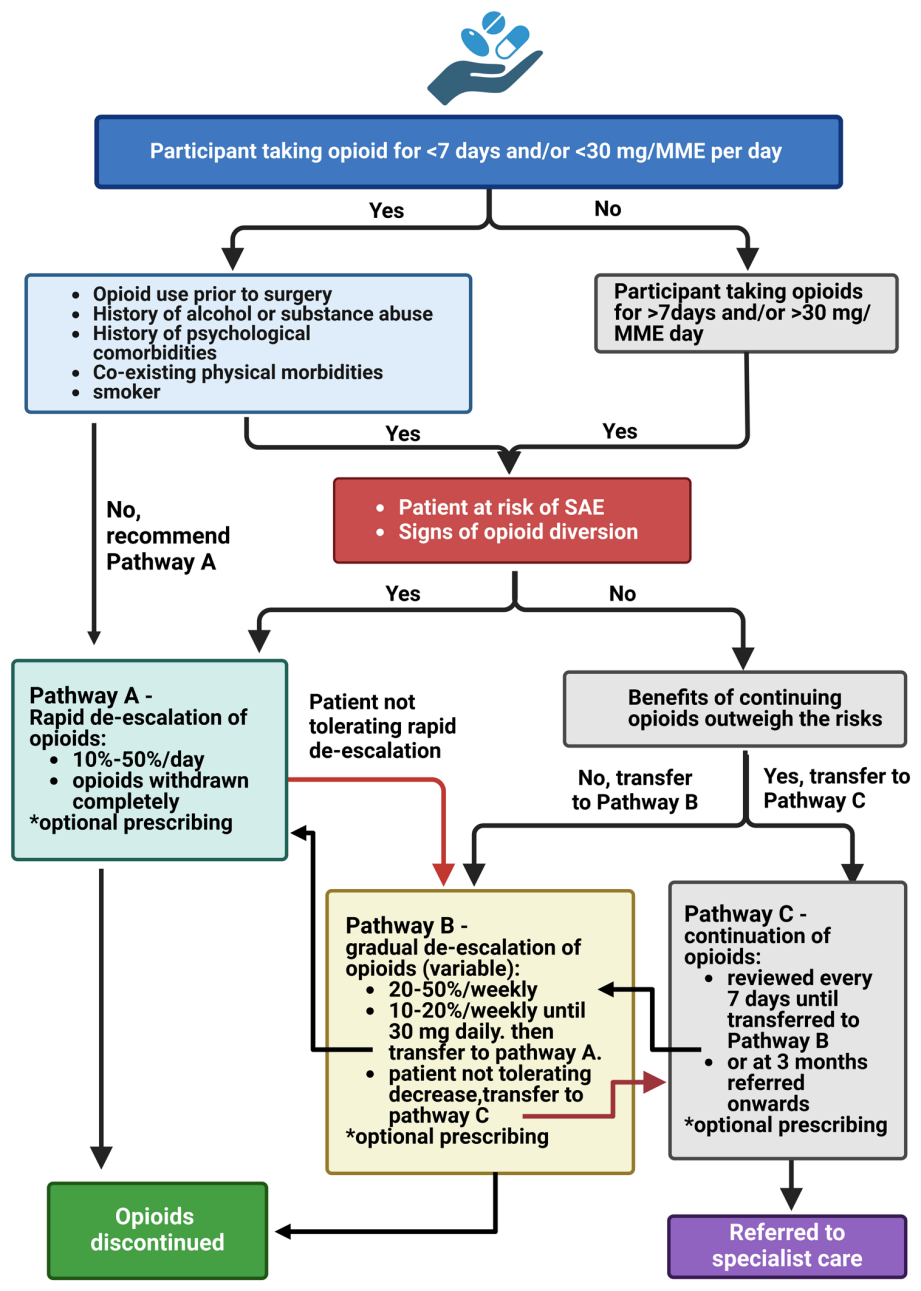
Intervention pathways and guidelines flow chart. N.B. These are just guidelines and subject to adjustment by the participating practices and pharmacists. *The pharmacist may also prescribe/recommend other pharmacological approaches to deal with: aches/pains – NSAIDs: Ibuprofen, 400mg/3X/daily or paracetamol, 1000 mg, every 6 hours; diarrhoea – Loperamide 2-4 mg as needed (up to 16 mg/day); constipation – laxative, as and when needed. Abbreviations: MME – morphine milligram equivalent; SAE – serious adverse event; hr – hour; mcg – microgram; NSAIDs – non-steroidal anti-inflammatory drugs. Image created by Biorender.com.

Following the MUR and biopsychosocial assessment, the clinical pharmacist will then decide with the patient which one of the three opioid de-escalation pathways (A, B or C) developed using various sources, American Pain Society ^41^, Veteran Affairs/Department of Defence ^42^, see also Kral *et al.*, 2015 ^43^ they will follow ( Figure 2). Due to a paucity of guidelines available for reference for dosage reduction or discontinuation of opioids particularly following surgery, guidance was adapted from the CDC Guideline for Prescribing Opioids for chronic pain ^44^; from the Veterans Health Administration Practice Guideline for Opioid Therapy in Chronic Pain ^45^; from the US Department of Health and Human Services Guide for Clinicians ^46^; and in collaboration with the local Community Chronic Pain Service.

### Pathway A – Rapid opioid de-escalation

For this pathway the clinical pharmacist may recommend immediate discontinuation of opioids if any of the following apply:

➢ Patient has been taking opioids for less than 7 days post-discharge and combined MME is less than < 30 mg/day (see Table 1).
➢ Opioids were not being used prior to surgery.
➢ Patient has no history of alcohol or substance abuse.
➢ The acute pain condition following surgery has resolved.
➢ The patient requests discontinuation of opioids.
➢ The patient has developed intolerable side effects from taking opioids.
➢ *The patient is at risk of a serious adverse event (SAE), such as overdose (refer to specialist service).
➢ *There is evidence of opioid diversion (refer to specialist service).

*Specialist services are required to manage these complex patients and will be referred to the Clinical Lead for guidance and recommendation.

### Pathway B – Gradual de-escalation of opioids

For this pathway the clinical pharmacist may recommend ‘gradual’ discontinuation of opioids if any of the following apply:

➢ Patient has been taking opioids for more than 7 days and/or the combined MME is more than > 30 mg – 120 mg/day or ≥12 µg/hr transdermal Fentanyl patches or ≥15 microgram (mcg)/hr Buprenorphine patches
➢ The medication is not providing useful pain relief and function has not improved
➢ Patient was taking opioids prior to surgery to treat pain related to their condition.
➢ Presents with other co-morbidities including mental health disorders
➢ Patient has a history of alcohol or substance abuse
➢ Patient is taking other medications (e.g., benzodiazepines, gabapentin, pregabalin or muscle relaxants) or medical conditions (lung disease, sleep apnoea, liver disease, kidney disease, fall risk, advanced age) that could increase the risk of adverse events (AEs)
➢ Patient requests a dosage reduction of their opioids
➢ The pharmacist feels it is safer to start with a gradual de-escalation of opioids

### Pathway C – Continuation of opioids

In some instances, the clinical pharmacist will recommend that the patient should continue taking their prescribed opioids, which is reviewed again after one week, if:

➢ Benefits of taking opioids outweigh the risk of discontinuing opioid therapy
➢ Prior opioid use was for another condition not related to the surgical procedure
➢ Patient does not want to discontinue their opioids

Pathway’s will not be exclusive to allow for patient variability in opioid withdrawal coping, and ongoing pain management and thus possible that patients will transition between pathway A and B, and from pathway C to B or A. We anticipate that for most participants, where opioids are being used primarily for the treatment of post-surgical pain or acute injury, pain which should normally subside after 3 – 4 days, and receiving opioids for the first time, will not require complex de-escalation protocols and psychological support. For those participants who have required prior (≤90 days) opioid use to manage their condition prior to surgery, or opioids for an unrelated condition, or on a higher dose, a more gradual discontinuation of opioids or referral to a specialist pain service, may be required.

### GP notification and medication change recommendations

The clinical pharmacist will record all medication changes and recommendations on the participants medical records. In cases where alternative medication or additional prescriptions are required this will be handled as per normal GP practice procedure.

### Withdrawal symptoms and adverse events

Due to the design of the intervention, we anticipate that withdrawal symptoms and adverse events that may occur as a result of opioid de-escalation will be minimal, and it at all will occur only in the first few hours or days. Any withdrawal symptoms or adverse events that are considered associated with opioid withdrawal will be recorded by the clinical pharmacist on the CRF. Any untoward medical occurrence that could be related to the study intervention will be recorded and reported to the study coordinator within 24 hrs. The Clinical Lead will review causality and if required report it to the sponsor (University of Kent) who will inform the NHS ethics committee.

### Outcomes measures

The primary outcome of the DESCALE study are to determine the feasibility of the designed intervention to support early deprescribing of opioids in this particular healthcare setting, using this particular healthcare personal and with this particular patient population, in order to inform a much larger, clinical trial. A summary of the study outcomes are listed in Table 3.

**Table 3.** Summary of outcome measures.

	Outcome Measure(s)
1	**Determination of** the fidelity of an early opioid deprescribing intervention led by trained clinical pharmacists in primary care and their role in the wider medicine’s optimisation programme
2	**Determination of** the barriers/enablers that affect the delivery of the intervention from the perspective of the NHS stakeholders
3	**Determination of** the barriers/enablers that affect the delivery of the intervention from the perspective of the surgical patient
4	**Establishment of** the cost of delivering the intervention from the health/social care provider (NHS), perspective
5	**Determination of** the percentage of participants that reduce or stop opioids within 91 days
6	**An understanding of** trends in surgical opioid prescribing, surgical related pain, and risk factors that contribute to long term opioid use
7	**Determination of** the integrity, robustness, and transferability of the protocol for future adoption by the NHS

### End of intervention

The end of the intervention is defined as when the participant has either discontinued their opioids and all final paperwork has been completed or after 3 months, where any participants still taking opioids at 91 days, and where pain will be considered chronic ^39^, will be referred on to specialist care.

### Data collection

Data variables collected pre-intervention or on day 0 will include baseline demographics, pain scores, EQ5D-5L (Quality of Life Questionnaire), and a number of key confounders that have been reported in the literature, and linked to long-term opioid use:

- Baseline demographics (age, sex, body mass index (BMI), ethnicity, social deprivation scores, smoking and alcohol consumption).
- Surgical history (surgery type, hospital, ward, opioid – type, strength, duration, average pain score).
- Medical history (prior opioid use, prior gabapentinoid and psychotropic use, other medication use, comorbidities, healthcare use).

At each additional follow-up appointment data variables collected will include pain scores, further medication changes, symptom management and for final appointments only, EQ5D-5L and Participant Feedback Questionnaire data. The aim of this questionnaire is to ascertain their experience of the deprescribing intervention, their views, and opinions on the process. In addition, all participants will be invited to take part in an online or telephone interview after their final follow-up with the pharmacist.

### Data confidentiality

All data recorded from participants will be de-identified to the research study team in line with NHS governance (Unique Reference Number (URN) and date of birth) and transmitted to a secure server which is confidentiality/privacy standard compliant. Access to the de-identified data will be accessible via a password-protected website, by authorised members of the research team. All participants in the study will be assigned a URN at the first MUR with the clinical pharmacist. This URN which will consist of GP practice initials followed by sequential number (double digits) and their date of birth will act as a medical identifier for each participant. All data collected from participants will be de-identified to the research study team using the URN.

### Data management

Submitted data will be collected, reviewed for completeness, analysed, and stored in compliance with the Data Protection Act of 2018 ^47^ by the University of Kent (UoK) research team members and public contributors where appropriate. All data will be entered onto a secure, database and accessible only to authorised members of the team. All participants recorded details will be anonymised and de-identified to the university’s research team by means of a unique research number, allocated during participant consent, by the clinical pharmacist. The handling of personal information by the research team will be clearly stated in the participant information sheet. All saved electronic data will be stored on password protected computers/laptops for a maximum of 5 years, post-project publication. All paper documentation, participant questionnaires, demographics data etc. will be stored in locked filing cabinets at the UoK (Medway School of Pharmacy). Paper documentation will be destroyed (shredded) five years post project publication. Similarly, all recorded interviews and transcripts will be deleted 5 years post-publication. Identifiable data (e.g., consent form) will be stored on secure UoK networks and destroyed 3 – 6 months after the study has ended.

### Statistical analysis

For the quantitative measures descriptive statistics will be used to describe the intervention population including patient demographic variables, deprivation scores, comorbidities for each surgical type, medication prescribed (including type, dose, amount, duration and long or short-acting), pain scores and history of opioid use. Descriptive statistics will also be used to assess de-escalation success rates, proportion of long-term users and quality of life. Similarly, descriptive statistics will be used to determine attrition rates at various stages and the different reasons provided. Means difference analyses will be performed via corrected repeated measures *t*-test/Wilcoxon’s tests or mixed ANOVAs (depending on data distribution and number of participants) to test the difference of MME taken at discharge and 91-days post discharge in each of the three pathways. The allocation to pathway C or not will be treated as a dichotomous outcome variable in a set of logistic regression models to determine the significance of a number of predictors as risk factors. Chi-square analyses will be used to compare the proportion of patients who accepted or declined the intervention. For the health economic analysis, a micro-costing approach will be used to estimate the intervention costs, opioid and other analgesic medication costs, and relevant participant health resource use. The qualitative data from the interviews will be transcribed verbatim, entered into qualitative data analysis software ( NVivo v11) or Microsoft Excel 2021 and subjected to thematic analysis ^48^. Notes collected at each medication assessment meeting will be reviewed and analyses for themes related to recommendations for the intervention and the role of clinical pharmacists in deprescribing opioids post-surgery.

### Ethics and amendments

The DESCALE feasibility study was granted ethical approval by the North-West Greater Manchester Central Research Ethics Committee on 19 ^th^ September 2023, reference number 23/NW/0241. The study will be conducted in accordance with the recommendations for physicians involved in research on human subjects adopted by the 18th World Medical Assembly, Helsinki 1964 (World Medical Association, 2013 ^49^, and later revisions) and the UK Policy Framework for Health and Social Care Research, 2022 ^50^. All data will be stored securely, and confidentiality of participants held in accordance with the Data Protection Act of 2018 ^47^. Any substantial amendments to the protocol or other study documents may require review and approval by the Research Ethics Committee (REC) before the changes can be implemented to the study. Where amendments are required, NHS Health Research Authority and REC procedures will be followed.

### Sponsorship and indemnity

This project is sponsored by the University of Kent (Reference: ResGov 470) who provide appropriate indemnity arrangements to cover research staff working on the project. The sponsorship contact is email: T.Coleman-581@kent.ac.uk. NHS indemnity covers the procedures carried out according to the prospective protocol at all NHS study GP sites.

### Audits

The study may be subject to audit by the University of Kent under their remit as sponsor and other regulatory bodies to ensure adherence to the UK Policy Framework for Health and Social Care Research.

### Sources of bias

All potential eligible participants will be approached using emails or SMS text and followed up by telephone and the intervention delivered mainly by video or telephone. This will introduce selection bias towards those participants who are technologically adept. In addition, by restricting eligibility to participants that are taking opioids less than 120 mg/MME day, we are biasing our selection towards potentially less medically complex patients.

### Study dissemination

The research findings will be disseminated as an accessible online report summarising key findings and recommendations, that will be distributed to all stakeholders and public groups supporting the study. Results will also be disseminated via an open access peer reviewed journal. Members of the research team may also disseminate findings through Stakeholder/University Twitter and LinkedIn accounts and at relevant third sector conferences.

### Study status

As of 23rd July 2024, three sites are currently active and have recruited 18 participants so far and one qualitative participant interview conducted.

### Strengths and limitations of this study

- This study will inform our understanding of patients’ experience of post-surgical opioid prescribing, surgical related pain and potentially identify individuals who require more support for opioid de-prescribing.
- This study will test the feasibility of an intervention designed to support patient and system outcomes in routine health settings, led by pharmacists.
- Patient data collected will provide a snapshot of real-world post-surgical prescribing in a single NHS trust, and highlight patient, medical and social factors that may influence long-term opioid dependence.
- This study will provide important information about current prescribing and deprescribing practices, however the small sample size (80–100) will limit the confidence in the statistical significance of these findings.
- Results on post-surgical opioid de-prescribing might not be generalisable to other- healthcare settings, professionals, or patient populations due to the localised study population.

## Discussion

Together with the local Chronic Pain Service, academics from the University of Kent, healthcare professionals from Kent Community Health NHS Foundation Trust and East Kent Hospital University Foundation Trust have come together to test an early deprescribing intervention working at the interface between secondary and primary care to determine whether the intervention is feasible and if it has the potential to prevent post-surgical patients transitioning from acute to long-term opioid persistent use. In addition, this study, combined with data collected as part of a parallel retrospective study looking at surgical prescribing data from the same trust, will provide information on perioperative opioid prescribing and the role it may play in contributing to opioid overuse in the community, for which there is a paucity of data in the UK.

## List of abbreviations

**AE:** Adverse Event

**ANOVA:** Analysis of Variance

**BMI:** Body Mass Index

**CRF:** Case Report Form

**CCG:** Clinical Commissioning Group

**DESCALE:** DE-eSCALation of opioid post-surgical discharge

**EKHUFT:** East Kent Hospitals University NHS Foundation Trust

**EQ5D-5L:** European Quality of Life 5 Dimension 5 Level

**GP:** General Practitioner

**hr:** hour

**HRA:** Health Research Authority

**mg:** milligram

**mcg:** microgram

**MME:** Morphine Milligram Equivalent

**MUR:** Medicines Use Review

**NHS:** National Health Service

**NIHR:** National Institute for Health and Care Research

**NSAID:** Non-Steroidal Anti-Inflammatory Drug

**PHE:** Public Health England

**PIL:** Patient Information Leaflet

**REC:** Research Ethics Committee

**SAE:** Serious Adverse Event

**SMS:** Standard Messaging Service

**SPIRIT:** Standard Protocol Items: Recommendation for International Trials

**UK:** United Kingdom

**UoK:** University of Kent

**URN:** Unique Reference Number

**US:** United States

## Ethics and consent

The DESCALE feasibility study was granted ethical approval by the North-West Greater Manchester Central Research Ethics Committee on 19 ^th^ September 2023, reference number 23/NW/0241. The study will be conducted in accordance with the recommendations for physicians involved in research on human subjects adopted by the 18th World Medical Assembly, Helsinki 1964 (World Medical Association, 2013 ^49^, and later revisions) and the UK Policy Framework for Health and Social Care Research, 2022 ^50^. All data will be stored securely, and confidentiality of participants held in accordance with the Data Protection Act of 2018 ^47^. Any substantial amendments to the protocol or other study documents may require review and approval by the Research Ethics Committee (REC) before the changes can be implemented to the study. Where amendments are required, NHS Health Research Authority and REC procedures will be followed.

Consent to participate in the study will be sought from all eligible patients that have undergone a surgical procedure and are discharged with an opioid prescription from the participating East Kent hospital sites and participating GP practices. Consent will only be sought after a full explanation and/or the PIL has been offered with time allowed for questions and proper consideration. Individuals willing to take part and for whom an MUR appointment has been made will be asked to complete a Participant Consent Form. A Consent form will be sent to those with digital capabilities prior to the MUR or completed together with the pharmacist at the first appointment. A typewritten signature or tick box declaration option, in accordance with the UK eIDAS Regulations (SI 2016/696) will be used for all digital consent forms. All consent forms will be checked and countersigned by the pharmacist prior to the commencement of the medication review. A copy of the consent form will be added to the participants medical records, and another retained in the study site folder, stored at the practice and a copy sent to the participant.

## Data Availability

Only the study team will have access to the full dataset throughout the study. A summary of findings will be reported to the participating research sites on completion of the study. Data sharing represents an efficient use of public money and supports more timely scientific discovery. Anonymised data will be made available after completion of this study to researchers at universities, NHS organisations or other healthcare providers where the sharing of data has a clearly defined purpose, and its use will be of benefit to wider society (Medical Research Council Policy and Guidance on Sharing of Research Data). **There are currently no data associated with this feasibility study.** Open Science Framework Data Repository: Pharmacist-led DE-eSCALation of opioids post-surgical dischargE (DESCALE), DOI 10.17605/OSF.IO/YV2CJ ^51^; URL: https://osf.io/yv2cj/ This project contains the following underlying data: Participant Consent Form - V3 - 05.09.2023.pdf Participant Information Leaflet - V3 - 05.09.2023.pdf Participant Satisfaction Questionnaire - V2 -11.05.2023.pdf SPIRIT_Fillable-checklist-15-Aug-2013 - 08.08.2024.doc Data are available under the terms of the Creative Commons Zero “No rights reserved” data waiver (CC0 1.0 Public domain dedication).

## Software availability

NVivo is a proprietary software, free alternatives such as Microsoft Excel ( https://www.microsoft.com/en-us/microsoft-365/excel) could be used.
