# Supplementary material for: Pharmacist-led DE-eSCALation of opioids post-surgical dischargE (DESCALE) – A multi-centre, non-randomised, feasibility study protocol
Source: NIHR Open Res. 2024 Aug 22;4:48. [Version 1] doi: 10.3310/nihropenres.13716.1 (PMC11757927; doi:10.3310/nihropenres.13716.1)
Supplement: Peer review history

# Reviewer report: Reviewer response for version 1

**DOI:** 10.3310/nihropenres.14892.r33042

**Published:** 2024-10-31

**Authors:** Liz Shaw

**Affiliations:** 1 University of Exeter, Exeter, England, UK

This protocol outlines a pharmacist-led programme to promote deprescribing of opioids post surgery. The methods propose a mixed-methods study.

 The protocol indicates that data collection has already commenced and I am uneasy about the retrospective publication of this protocol. It limits the potential utility of my comments and the ability of the study authors to address comments relevant to improving the conduct of the study and somewhat undermines the peer-review process. I hope the queries and comments below are helpful to the authors in refining their reporting of their methods.

 Further details regarding methods:

 Intervention Population:

 1) Further rationale for the study population would be useful. Is it appropriate to implement the intervention at same time point for different surgery populations? i.e. do recovery timeframes differ? Are there some operations where it may be realistically expected for recovery to be longer? Will some age groups be expected to have longer recovery/need opiates for longer? This may be covered by the exclusion criteria stating max. opioid dose and Pathway C but could be made more explicit.

 Recruitment:

 2) My comments here pertain mainly to study methods. Patients may be more able/willing to consent if contacted prior to recruitment. Could imagine some would be most unwilling after a hospital admission to consider a surprise research participation request after being discharged home. Online consent form will not be accessible to some populations (Lower SES, older people, not technologically literate). Consent form in person with Pharmacist may place some under pressure to participate. How can potential participants be given a longer time to consider if they wish to participate and identify questions they may have?

 Data Collection

 3) For qualitative interviews - Why only patient participants? This intervention has implications for family, carers, pharmacists and other healthcare professionals (in both primary and secondary care). To fully assess feasibility and acceptability, rich qualitative data from these groups would also be extremely beneficial.

 4) Further detail regarding qualitative interviews required - Interview schedule, Where will they be conducted? Duration?

 Outcomes

 5) It is not clear which outcome measures will be used to measure which outcomes at which timepoints. Further clarity here would be useful.

  6) Will long term PROMS/PREMS be collected post 3-month discharge (see point 11 below)? This may enable triangulation with qualitative data.

 7) How will authors consider impact of potential health inequities in their data collection and analyses (e.g. those posited by PROGRESS-PLUS criteria)

 8) Will hospital readmissions (both under and over 30 days post discharge) be assessed alongside use of primary care services to see if intervention has any unintended negative consequences for service use?

 9) For the outcomes in Table 3: How will these be measured?

 10) Should a measure of anxiety/depression e.g. PHQ-9 or HADS be used to confirm MH diagnosis and patient current level of symptomology (and allow baseline-post intervention comparisons)? Should patient self-harm/suicide risk be monitored and how will this be dealt with should concerns arise?

 Study timelines:

 11) Is it feasible to conduct longer term follow up? Patient reported outcome measures and patient reported experience measures post-hospital discharge are rarely assessed in the long-term, as is the case here, which limits conclusions that can be drawn re: safety/efficacy/acceptability of an intervention.

 Analysis

 12) How will authors take into account influence of different populations/procedures and consider impact of possible health inequities (e.g. those detailed by PROGRESS-PLUS criteria) across analysis of both quant and qual data.

 13) Further detail on rationale for analysis methods chosen and methods would be helpful. Why is thematic analysis of interview data most suited method?

 How will you reduce risk of bias in your analysis e.g. double coding, checking arising themes with PPI/HCP stakeholders?

 Will the questions you ask interviewees remain fixed throughout study, or will the interview schedule evolve - informed by the content/analysis of previous interviews (a more Grounded Theory Approach)

 Will you make any attempts to integrate quantitative and qualitative findings? If so, how?

 14) I am not best placed to comment on methods used to calculate costs, but I think further details are required re: calculating your cost data. E.g. What factors are you you considering? What sources for costs are you using?

Is the study design appropriate for the research question?

Yes

Is the rationale for, and objectives of, the study clearly described?

Yes

Are sufficient details of the methods provided to allow replication by others?

No

Are the datasets clearly presented in a useable and accessible format?

No

Reviewer Expertise:

Systematic reviewer; expertise in qualitative synthesis

I confirm that I have read this submission and believe that I have an appropriate level of expertise to confirm that it is of an acceptable scientific standard, however I have significant reservations, as outlined above.

# Response

**Published:** 2025-01-07

**Authors:** Emma Veale

**Affiliations:** Pharmacy, University of Kent Division of Natural Sciences, Canterbury, England, UK

Reviewer Comments:

 This protocol outlines a pharmacist-led programme to promote deprescribing of opioids post-surgery. The methods propose a mixed-methods study.

 The protocol indicates that data collection has already commenced and I am uneasy about the retrospective publication of this protocol. It limits the potential utility of my comments and the ability of the study authors to address comments relevant to improving the conduct of the study and somewhat undermines the peer-review process. I hope the queries and comments below are helpful to the authors in refining their reporting of their methods.

***Author Response:***

***Dear Dr Shaw, thank you for reviewing our protocol and for your helpful comments and suggestions. We have responded to your comments below in bold and in the manuscript.***

***It is not uncommon when publishing protocol papers of this type that the study has already commenced. The only requirement is that it is not submitted once data collection has finished. A protocol of this type will already have undergone multiple rounds of funding external peer review and then additional rounds of review by NHS ethics, including an interview, prior to the intervention commencing and additionally any amendments to the protocol approved via NHS ethics committee, as the study commences. The data we subsequently collect will allow us to report on whether this protocol is feasible or not, how it can be improved, so that it can be adopted in the field or equally, any pitfalls we uncover can be avoided by others.***

 Reviewer Comments:

 Further details regarding methods:

 Intervention Population:

 1) Further rationale for the study population would be useful. Is it appropriate to implement the intervention at same time point for different surgery populations? i.e. do recovery timeframes differ? Are there some operations where it may be realistically expected for recovery to be longer? Will some age groups be expected to have longer recovery/need opiates for longer? This may be covered by the exclusion criteria stating max. opioid dose and Pathway C but could be made more explicit.

***Author Response:***

***These are very good points and the type of things we hope to determine from the data collected during the feasibility study. We have offered 3 pathways (rapid, slower, no change) which we hope will allow flexibility for just these points you have raised. We have added more description to pathway C to reflect this.***

 Reviewer Comments:

 Recruitment:

 2) My comments here pertain mainly to study methods. Patients may be more able/willing to consent if contacted prior to recruitment. Could imagine some would be most unwilling after a hospital admission to consider a surprise research participation request after being discharged home. Online consent form will not be accessible to some populations (Lower SES, older people, not technologically literate). Consent form in person with Pharmacist may place some under pressure to participate. How can potential participants be given a longer time to consider if they wish to participate and identify questions they may have?

***Author Response:***

***Yes, we totally agree. Our initial hope was to recruit directly from surgical wards; and use a team of study pharmacists to deliver the intervention. However, we realised quite early on as we designed the protocol that pharmacists would need to be affiliated to a GP practice in order to access medical records and screening patients on a hospital ward or from a hospital pharmacy would be untenable for the staff involved. We considered that patients receiving an email or text message directly from their GP surgery would be something that they are accustomed to, rather than a surprise.***

***We totally accept that online consent is only accessible to those with digital capabilities, although we can offer to take them through a consent form over the telephone and we do offer telephone MURs. Unfortunately, due to the timeframe which we want to deliver these MURS we were unable to offer postal options. We accept that this is a limitation of the study, and we noted this in the sources of bias section of the manuscript. A consent form is only completed with the patient and appointment made with the pharmacist if they agree to take part after consideration. No patient is coerced into taking part by the practice staff.***

 Reviewer Comments:

 Data Collection

 3) For qualitative interviews - Why only patient participants? This intervention has implications for family, carers, pharmacists and other healthcare professionals (in both primary and secondary care). To fully assess feasibility and acceptability, rich qualitative data from these groups would also be extremely beneficial.

***Author Response:***

***Thank you for pointing this out. Qualitative interviews are intended for all those that participated, including HCPs. We also intend to interview key stakeholders from the ICB and from EKHUFT. We have amended the text to make this clear and added a section on HCP feedback that was inadvertently missing.***

 Reviewer Comments:

 4) Further detail regarding qualitative interviews required - Interview schedule, Where will they be conducted? Duration?

***Author Response:***

***Thank you for highlighting this. We have amended the text to provide more detail about these interviews.***

 Reviewer Comments:

 Outcomes

 5) It is not clear which outcome measures will be used to measure which outcomes at which timepoints. Further clarity here would be useful.

***Author Response:***

***We have combined the outcomes with the aims and objectives table (1) so that this is clearer to the reader.***

 Reviewer Comments:

  6) Will long term PROMS/PREMS be collected post 3-month discharge (see point 11 below)? This may enable triangulation with qualitative data.

***Author Response:***

***Due to the budget and timeframe, we are regrettably unable to collect long term PROMS/PREMS data for this study. Should we be able to conduct a larger study, then this is something that we would want to do.***

 Reviewer Comments:

 7) How will authors consider impact of potential health inequities in their data collection and analyses (e.g. those posited by PROGRESS-PLUS criteria)

***Author Response:***

***We are collecting and will report socioeconomic factors of participants in this study. However, the small sample size will make any inferences of this data limited.***

 Reviewer Comments:

 8) Will hospital readmissions (both under and over 30 days post discharge) be assessed alongside use of primary care services to see if intervention has any unintended negative consequences for service use?

***Author Response:***

***Any adverse events whether related to the intervention or not must be reported by the practice to the study team. Due to this intervention designed to target mostly opioid naïve participants on ≤120 mg MME/day we anticipate few if any unintended negative consequences to occur.***

 Reviewer Comments:

 9) For the outcomes in Table 3: How will these be measured?

***Author Response:***

***We have recombined these with the aims and objectives (Table 1), so that this is clearer to the reader.***

 Reviewer Comments:

 10) Should a measure of anxiety/depression e.g. PHQ-9 or HADS be used to confirm MH diagnosis and patient current level of symptomology (and allow baseline-post intervention comparisons)? Should patient self-harm/suicide risk be monitored and how will this be dealt with should concerns arise?

***Author Response:***

***As for question 8, the intervention targets patients that are early on in an opioid journey and do not have a previous history of long-term opioid or drug abuse. Therefore, we do not anticipate any serious adverse events relating to opioid deescalation. The CRF however captures all medication use by the participant and any red flags should be recognised by the pharmacists who are trained to deliver high risk MURs and reported to the clinical lead who has also vast experience of working with long-term opioid dependent patients.***

 Reviewer Comments:

 Study timelines:

 11) Is it feasible to conduct longer term follow up? Patient reported outcome measures and patient reported experience measures post-hospital discharge are rarely assessed in the long-term, as is the case here, which limits conclusions that can be drawn re: safety/efficacy/acceptability of an intervention.

***Author Response:***

***We agree with you completely, but for this small project it has not been possible. We would hope to address this in a larger study. The purpose of this study is to determine whether it is feasible, what needs to improve and what type of patient we should target or not target, with this type of intervention.***

 Reviewer Comments:

 Analysis

 12) How will authors take into account influence of different populations/procedures and consider impact of possible health inequities (e.g. those detailed by PROGRESS-PLUS criteria) across analysis of both quant and qual data.

 13) Further detail on rationale for analysis methods chosen and methods would be helpful. Why is thematic analysis of interview data most suited method?

 How will you reduce risk of bias in your analysis e.g. double coding, checking arising themes with PPI/HCP stakeholders?

 Will the questions you ask interviewees remain fixed throughout study, or will the interview schedule evolve - informed by the content/analysis of previous interviews (a more Grounded Theory Approach)

 Will you make any attempts to integrate quantitative and qualitative findings? If so, how?

 14) I am not best placed to comment on methods used to calculate costs, but I think further details are required re: calculating your cost data. E.g. What factors are you you considering? What sources for costs are you using?

***Author Response:***

***We have added more detail around the methods and statistical analysis in the manuscript to address your comments in questions 12, 13 and 14 and to provide more clarity.***

# Reviewer report: Reviewer response for version 1

**DOI:** 10.3310/nihropenres.14892.r33044

**Published:** 2024-10-25

**Authors:** Neetu Bansal

**Affiliations:** 1 The University of Manchester, Manchester, England, UK

Thank you for the opportunity to review this submission, which addresses the important issue of problematic long-term opioid use in post-surgical patients. The mixed-methods approach employed in this feasibility study is well thought out and pragmatic, making it suitable for exploring the practicalities of early opioid de-escalation in primary care settings.

 There are several strengths in the study's aims and objectives, particularly in addressing a key public health issue. However, there are also a few areas that would benefit from further clarification and refinement to enhance the study’s overall impact and feasibility. Below are some points for consideration:

  

1. Some objectives, such as "ascertain stakeholder acceptability of the intervention" and "ascertain participant acceptability of the intervention," are somewhat broad and vague. These could be more clearly defined by specifying the exact dimensions of acceptability that will be measured, such as willingness to participate, perceived effectiveness, or identification of barriers to implementation.

- While stakeholder interviews are mentioned, there is no clear identification of who the stakeholders are (e.g., GPs, surgeons, pharmacists, patients). Clarifying the stakeholder groups and how their perspectives will be incorporated into the analysis would strengthen the study. It would also be helpful to describe how the results from these interviews will influence the development or adaptation of the intervention
- The objective to "Measure the capabilities of trained clinical pharmacists" references "observational competency checks by the Clinical Lead," but there is no information on how these checks will be standardised across different pharmacists or validated. Providing more detail on how competency will be measured (e.g., using competency frameworks or objective performance metrics) would enhance the robustness of this objective. Additional information on the training modules used to assess pharmacist capabilities would also be useful.

- The use of EQ-5D-5L for measuring quality of life and pain scores is appropriate, but there is no mention of how changes in these metrics will be analysed in relation to opioid de-escalation. Outlining a plan for analysing pain and quality of life data in relation to opioid use (e.g., through subgroup analyses) would strengthen the interpretation of outcomes

- There is no clear plan for managing participants who do not respond to the intervention or who drop out before completion. Including an objective or process for handling non-responders would provide valuable insights into barriers to intervention effectiveness and inform the design of future studies.

**Additional Considerations:**

- **Introduction**: The statement that opioids cause "more pain" needs clarification. It would be helpful to explicitly reference opioid-induced hyperalgesia to clarify that you are referring to increased sensitivity to pain as a potential side effect of long-term opioid use.
- **Methods**:
  - It is unclear how often the discharge list will be generated and what the process will be for identifying patients. Providing more detail on how discharge lists will be compiled and reviewed would improve the transparency of participant identification.
  - What is the level of buy-in from clinical pharmacists? Will the same pharmacist consistently see each patient during follow-up? Consistency in pharmacist-patient interaction could influence outcomes, so it would be helpful to clarify this aspect.
  - Clarification is needed regarding the types of surgical procedures included in the study. Will both minor and major surgeries be included, and will day-case procedures also be part of the intervention?
  - Exclusion criteria - how will you decide on 'severe' co-existing medical condition (using standardised co-morbidity scoring systems for example)
  - Pathway C - refer to non-pharmacological options as an option?
  - Pathway C - Continuation of opioids,  benefits of taking opioids outweigh the risk of discontinuing opioid therapy - can you give examples of such situations as this appears vague
  - Recruitment period Jan to September 2014. States 17 participants recruited. Does this refer to the patients? As per protocol sample size to aim for is around 100 participants.

In summary, this is a well-constructed study that addresses an important topic. Strengthening the clarity and detail in the areas highlighted above would enhance the study’s feasibility and robustness, ensuring it provides actionable insights into the effectiveness of early opioid de-escalation.

Is the study design appropriate for the research question?

Partly

Is the rationale for, and objectives of, the study clearly described?

Yes

Are sufficient details of the methods provided to allow replication by others?

Partly

Are the datasets clearly presented in a useable and accessible format?

Not applicable

Reviewer Expertise:

Pain management in surgical patients and medicines optimisation

I confirm that I have read this submission and believe that I have an appropriate level of expertise to confirm that it is of an acceptable scientific standard, however I have significant reservations, as outlined above.

# Response

**Published:** 2025-01-07

**Authors:** Emma Veale

**Affiliations:** Pharmacy, University of Kent Division of Natural Sciences, Canterbury, England, UK

Thank you for the opportunity to review this submission, which addresses the important issue of problematic long-term opioid use in post-surgical patients. The mixed-methods approach employed in this feasibility study is well thought out and pragmatic, making it suitable for exploring the practicalities of early opioid de-escalation in primary care settings.

***Dear Dr Bansal, thank you for reviewing the manuscript, for your positive comments and recommendations to improve it. We realise that in an attempt to be concise we have missed some important details. We have responded to your queries below in bold and in the edited manuscript.***

 There are several strengths in the study's aims and objectives, particularly in addressing a key public health issue. However, there are also a few areas that would benefit from further clarification and refinement to enhance the study’s overall impact and feasibility. Below are some points for consideration:

  

1. Some objectives, such as "ascertain stakeholder acceptability of the intervention" and "ascertain participant acceptability of the intervention," are somewhat broad and vague. These could be more clearly defined by specifying the exact dimensions of acceptability that will be measured, such as willingness to participate, perceived effectiveness, or identification of barriers to implementation.

- While stakeholder interviews are mentioned, there is no clear identification of who the stakeholders are (e.g., GPs, surgeons, pharmacists, patients). Clarifying the stakeholder groups and how their perspectives will be incorporated into the analysis would strengthen the study. It would also be helpful to describe how the results from these interviews will influence the development or adaptation of the intervention

***We agree, these objectives are vague and concede that we have inadvertently oversimplified Table1 in an attempt to be concise. We have clarified the stakeholder groups that will be approached and added more detail around what information will be collected to inform the design of a future intervention in Table 1 and in the manuscript.***

  

- The objective to "Measure the capabilities of trained clinical pharmacists" references "observational competency checks by the Clinical Lead," but there is no information on how these checks will be standardised across different pharmacists or validated. Providing more detail on how competency will be measured (e.g., using competency frameworks or objective performance metrics) would enhance the robustness of this objective. Additional information on the training modules used to assess pharmacist capabilities would also be useful.

***Response: We determined that this type of MUR being delivered was standard procedure for the pharmacists participating in the study (most had been involved in a high-use opioid study previously with the clinical lead), and patients recruited deemed low-risk compared to long-term users of opioids. Therefore, the competency would be as expected in undertaking a standard MUR. However, all pharmacists received additional training by the Clinical Lead to conduct the MUR for the purpose of the study. For this they received an hour-long opioid lecture at the start, with signposting to multiple learning resources and regular individual contact with Dr Theron on each patient’s reduction programme. For this feasibility study, we designed broad reduction pathways options (A, B, C,) for the pharmacists to follow and weekly drop-in sessions with the Clinical Lead for pharmacists to discuss any clinical queries that may have arisen. Each pharmacist also has a responsible GP to check their prescriptions as part of the partaking surgeries’ agreement. Fidelity checks of the filled CRFs are performed by the research team and during the Clinical Lead’s drop-in sessions for the pharmacists. We have replaced the word measure with assess. It is possible that more in-depth training in the form of modules may be a finding of our qualitative interviews with participating pharmacists.***

  

- The use of EQ-5D-5L for measuring quality of life and pain scores is appropriate, but there is no mention of how changes in these metrics will be analysed in relation to opioid de-escalation. Outlining a plan for analysing pain and quality of life data in relation to opioid use (e.g., through subgroup analyses) would strengthen the interpretation of outcomes

***Thank you for pointing this out. We have added a bit more information as to how we will use this data in the analysis section.***

- There is no clear plan for managing participants who do not respond to the intervention or who drop out before completion. Including an objective or process for handling non-responders would provide valuable insights into barriers to intervention effectiveness and inform the design of future studies.

***The practices collect baseline data (age, gender and surgical procedure) of all participants eligible for the intervention on the recruitment log. This data will be used to determine whether these factors influence non/participation in the study. We have tried to make this clearer in the objectives and participant recruitment section. Unfortunately, due to the short time frame between identifying participants and inviting them to the study, we can only approach eligible participants once.***

**Additional Considerations:**

- **Introduction**: The statement that opioids cause "more pain" needs clarification. It would be helpful to explicitly reference opioid-induced hyperalgesia to clarify that you are referring to increased sensitivity to pain as a potential side effect of long-term opioid use.

***Thank you for pointing this out. It had slipped in from previous lay summaries of the research. We have amended this***.

- **Methods**:
  - It is unclear how often the discharge list will be generated and what the process will be for identifying patients. Providing more detail on how discharge lists will be compiled and reviewed would improve the transparency of participant identification.

***We had written under recruitment, that searches would be undertaken on a weekly basis, however this would be at the discretion and capacity of the practice and pharmacists to do this. I have adjusted to say weekly/bi-monthly as this is more realistic of what we are experiencing. I agree that the method used to search for eligible patients is lacking. This is because individual GP practices have had to develop searches based on the systems that they use and the way in which they handle the discharge lists. No practice has been the same and will be reported as one of the barriers!***

  

-
  - What is the level of buy-in from clinical pharmacists? Will the same pharmacist consistently see each patient during follow-up? Consistency in pharmacist-patient interaction could influence outcomes, so it would be helpful to clarify this aspect.

***We will capture the level of buy-in from pharmacists through the interview process. We intend to interview pharmacists that engaged and those that didn’t engage at practices which had undergone site-initiation. Time permitting, we will also approach pharmacists from other practices that had initially expressed an interest but did not participate. I have added this to the objectives.***

***We did not stipulate that the same pharmacist must complete follow-up reviews with the same participate. We have left this decision to the discretion of the practice. However, as the pharmacist books the next appointment with the patient at the MUR and most practices only have one pharmacist participating this has occurred spontaneously.***

  

-
  - Clarification is needed regarding the types of surgical procedures included in the study. Will both minor and major surgeries be included, and will day-case procedures also be part of the intervention?

***We include all types of surgical procedure (inpatient and outpatient), with the exclusion of caesarean sections and cancer patients. We have amended the inclusion criteria to be clearer.***

-
  - Exclusion criteria - how will you decide on 'severe' co-existing medical condition (using standardised co-morbidity scoring systems for example)

***No scoring is done, the rationale used will be the same as in any other clinic situation. Where there is uncertainty about a participant’s suitability, this is discussed directly with the Clinical Lead who is the lead Clinician of a Chronic Pain service. If during the time of the project that assessing the severity of a medical condition is proving difficult for pharmacists, then we would report this as a barrier and use this information to adapt the intervention going forward. We have amended the exclusion criteria to reflect this.***

-
  - Pathway C - refer to non-pharmacological options as an option?

***This is not an option of this pathway or the project and would relate more to patients that are suffering from chronic pain (>91 days) conditions or those that continue with opioids past 90 days and who are transferred back into the system for support.***

-
  - Pathway C - Continuation of opioids,  benefits of taking opioids outweigh the risk of discontinuing opioid therapy - can you give examples of such situations as this appears vague

***This relates to the pain scores, where the patient is still suffering from severe pain at the time of the MUR which would warrant a continuation of opioids and in some circumstances potentially an increase in dose or a change in medication. We have amended the text to reflect this.***

-
  - Recruitment period Jan to September 2014. States 17 participants recruited. Does this refer to the patients? As per protocol sample size to aim for is around 100 participants.

***Yes, this related to the number of patients recruited at the time of submission of the manuscript. However, it should read 2024, not 2014!***

 In summary, this is a well-constructed study that addresses an important topic. Strengthening the clarity and detail in the areas highlighted above would enhance the study’s feasibility and robustness, ensuring it provides actionable insights into the effectiveness of early opioid de-escalation.

***Thank you!***

# Reviewer report: Reviewer response for version 1

**DOI:** 10.3310/nihropenres.14892.r32791

**Published:** 2024-09-16

**Authors:** Judy Mullan

**Affiliations:** 1 University of Wollongong, Wollongong, New South Wales, Australia

Thank you for the opportunity to review this submission, which addresses the critical issue of long-term opioid use. The rationale for the study is clearly articulated, emphasising that while opioids can be prescribed for short-term pain management after surgery, their long-term use can lead to dependence, overdose, and even death in severe cases. This is a significant public health concern worldwide. Reducing long-term opioid use among post-surgical patients would therefore help address an important global public health issue.

 The study aims to fill a gap in the current literature by identifying an effective, evidence-based strategy using trained clinical pharmacists in GP practices to reduce long-term opioid use in post-surgical patients. Additionally, it seeks to assess the feasibility of the pharmacist-led intervention. Evaluating the feasibility of this intervention—including ease of implementation, acceptance by healthcare providers and patients, and establishing its effectiveness—can provide valuable insights for implementation on a much broader scale and would also help to inform policy and future practice.

 The study design, a prospective multi-centre, non-randomised pragmatic feasibility sub-study within the DESCALE study is appropriate to answer the research questions and the methods provided allow for replication by others. However, there are some points which need to be clarified regarding some of the objectives and data collection points for the different aims in Table 1(Summary of Aims and Objectives):

 For example:

 Aim/question 1: To test the feasibility of delivering an early opioid deprescribing intervention in primary care, lead by trained clinical pharmacist

 -To ascertain stakeholder acceptability – it is not clear where the detailed records of any barriers or enablers in response to setting up and whilst delivering the intervention will be drawn from. It is also not clear which stakeholders and how many will be interviewed

 In addition, more clarity is needed regarding the modules which will be used to measure the capabilities of the trained clinical pharmacists. Have they already been developed or are they going to be developed as part of the study?

 In addition to measuring acceptability of the intervention by measuring patient participant engagement would it also be worthwhile to measure health practitioner engagement?

 Aim/question: To understand post-surgical opioid prescribing experiences including opioid use, long-term opioid use, participant pain and quality of life in the 3-month period post-surgery when discharged with an opioid prescription

 It is not clear what the research team means by “Responses during participant interviews.”  Does this mean that it is the intention to interview all study participants or a subset of the study participants?

 Aim/questions: To ascertain the feasibility of delivering the intervention as per the protocol to inform a future study or adoption of the protocol?

 The protocol suggests that measurements will include a number of process and outcome measures which are indeed important. However, to better understand the feasibility of conducting an intervention the following questions would also need to be addressed:

-
  - Acceptability -from the perspective of the patients and the healthcare providers
  - what are the required resources (time, personnel, equipment) for implementing the protocol.
  - What are the barriers and facilitators to implementation of the intervention and is it practical.
  - How easily can the intervention be integrated in the existing health care system
  - Will the intervention need to be adapted for different primary care/clinical settings
  - What are the potential challenges in scaling the intervention and what resources are required to sustain the intervention in the long-term?

While the outcome measures are identified in Table 3 (Summary of study outcomes) it is not clear in the submitted protocol how the data for some of these outcomes will be collected and measured.

 In particular

- Determination of the barriers/enablers that affect the delivery of the intervention from the perspective of the NHS stakeholders
- Determination of the barriers/enablers that affect the delivery of the intervention from the perspective of the
- Determination of the integrity, robustness, and transferability of the protocol for future adoption by the NHS

 

 Thank you for the opportunity to review this submission, which addresses the critical issue of long-term opioid use. The rationale for the study is clearly articulated, emphasising that while opioids can be prescribed for short-term pain management after surgery, their long-term use can lead to dependence, overdose, and even death in severe cases. This is a significant public health concern worldwide. Reducing long-term opioid use among post-surgical patients would therefore help address an important global public health issue.

 The study aims to fill a gap in the current literature by identifying an effective, evidence-based strategy using trained clinical pharmacists in GP practices to reduce long-term opioid use in post-surgical patients. Additionally, it seeks to assess the feasibility of the pharmacist-led intervention. Evaluating the feasibility of this intervention—including ease of implementation, acceptance by healthcare providers and patients, and establishing its effectiveness—can provide valuable insights for implementation on a much broader scale and would also help to inform policy and future practice.

 The study design, a prospective multi-centre, non-randomised pragmatic feasibility sub-study within the DESCALE study is appropriate to answer the research questions and the methods provided allow for replication by others. However, there are some minor points which could be clarified regarding some of the objectives and data collection points for the different aims in Table 1(Summary of Aims and Objectives):

 Aim/question 1: To test the feasibility of delivering an early opioid deprescribing intervention in primary care, lead by trained clinical pharmacist

 -To ascertain stakeholder acceptability – it is not clear where the detailed records of any barriers or enablers in response to setting up the intervention and whilst delivering the intervention will be drawn from. It is also not clear which stakeholders and how many will be interviewed

 It is also not clear if the modules to measure the capabilities of trained clinical pharmacists have already been developed or are to be developed as part of the study

 In addition to measuring participant acceptability of the intervention by measuring patient participant engagement would it also be worthwhile to measure health practitioner engagement?

 Aim/ question: To understand post-surgical opioid prescribing experiences including opioid use, long-term opioid use, participant pain and quality of life in the 3-month period post-surgery when discharged with an opioid prescription

 It is not clear what the research team means by “Responses during participant interviews.”  Does this mean that it is the intention to interview all study participants or a subset of the study participants?

 Aim/questions: To ascertain the feasibility of delivering the intervention as per the protocol to inform a future study or adoption of the protocol?

 The protocol suggest that measurements will include a number of process measurements and outcome measures which are indeed important. However, to better understand the feasibility of conducting an intervention the following questions would also need to be addressed:

-
  - Acceptability -from the perspective of the patients and the healthcare providers
  - what are the required resources (time, personnel, equipment) for implementing the protocol.
  - What are the barriers and facilitators to implementation of the intervention and is it practical.
  - How easily can the intervention be integrated in the existing health care system
  - Will the intervention need to be adapted for different primary care/clinical settings
  - What are the potential challenges in scaling the intervention and what resources are required to sustain the intervention in the long-term?

While the outcome measures are clearly identified in Table 3 (Summary of study outcomes) it is not clear in the submitted protocol how the data for some of these outcomes will be collected and measured.

 In particular

- Determination of the barriers/enablers that affect the delivery of the intervention from the perspective of the NHS stakeholders
- Determination of the barriers/enablers that affect the delivery of the intervention from the perspective of the
- Determination of the integrity, robustness, and transferability of the protocol for future adoption by the NHS

Is the study design appropriate for the research question?

Partly

Is the rationale for, and objectives of, the study clearly described?

Yes

Are sufficient details of the methods provided to allow replication by others?

Partly

Are the datasets clearly presented in a useable and accessible format?

Not applicable

Reviewer Expertise:

Quality Use of Medicines

I confirm that I have read this submission and believe that I have an appropriate level of expertise to confirm that it is of an acceptable scientific standard, however I have significant reservations, as outlined above.

# Response

**Published:** 2025-01-07

**Authors:** Emma Veale

**Affiliations:** Pharmacy, University of Kent Division of Natural Sciences, Canterbury, England, UK

Reviewer 1 – Judy Mullan

***Dear Dr Mullan, thank you for reviewing the manuscript, for your positive comments and recommendations to improve it. We have responded to your comments below and amended the manuscript accordingly. Best wishes, DESCALE team.***

 Thank you for the opportunity to review this submission, which addresses the critical issue of long-term opioid use. The rationale for the study is clearly articulated, emphasising that while opioids can be prescribed for short-term pain management after surgery, their long-term use can lead to dependence, overdose, and even death in severe cases. This is a significant public health concern worldwide. Reducing long-term opioid use among post-surgical patients would therefore help address an important global public health issue.

 The study aims to fill a gap in the current literature by identifying an effective, evidence-based strategy using trained clinical pharmacists in GP practices to reduce long-term opioid use in post-surgical patients. Additionally, it seeks to assess the feasibility of the pharmacist-led intervention. Evaluating the feasibility of this intervention—including ease of implementation, acceptance by healthcare providers and patients, and establishing its effectiveness—can provide valuable insights for implementation on a much broader scale and would also help to inform policy and future practice.

 The study design, a prospective multi-centre, non-randomised pragmatic feasibility sub-study within the DESCALE study is appropriate to answer the research questions and the methods provided allow for replication by others. However, there are some points which need to be clarified regarding some of the objectives and data collection points for the different aims in Table 1(Summary of Aims and Objectives):

 For example:

 Aim/question 1: To test the feasibility of delivering an early opioid deprescribing intervention in primary care, lead by trained clinical pharmacist

 -To ascertain stakeholder acceptability – it is not clear where the detailed records of any barriers or enablers in response to setting up and whilst delivering the intervention will be drawn from.

***Regarding your query around barriers and enablers. We anticipate that these will arise from all aspects of the study delivered – from recruitment, participation, delivery of the MURs, clinical drop-in sessions, stakeholder queries, to the collection of the data and from the interviews. All of these are being recorded as the study progresses.***

 It is also not clear which stakeholders and how many will be interviewed

***Thank you for highlighting that this was missing from the manuscript. We are indeed interviewing stakeholders and have adding in the information that was missing to the manuscript.***

 In addition, more clarity is needed regarding the modules which will be used to measure the capabilities of the trained clinical pharmacists. Have they already been developed or are they going to be developed as part of the study?

 R ***esponse: We determined that this type of MUR being delivered was standard procedure for the pharmacists participating in the study (most had been involved in a high-use opioid study previously with the clinical lead), and patients recruited deemed low-risk compared to long-term users of opioids. Therefore, the competency would be as expected in undertaking a standard MUR. However, all pharmacists received additional training by the Clinical Lead to conduct the MUR for the purpose of the study. For this they received an hour-long opioid lecture at the start, with signposting to multiple learning resources and regular individual contact with Dr Theron on each patient’s reduction programme. For this feasibility study, we designed broad reduction pathways options (A, B, C,) for the pharmacists to follow and weekly drop-in sessions with the Clinical Lead for pharmacists to discuss any clinical queries that may have arisen. Each pharmacist also has a responsible GP to check their prescriptions as part of the partaking surgeries’ agreement. Fidelity checks of the filled CRFs are performed by the research team and during the Clinical Lead’s drop-in sessions for the pharmacists. We have added in more information to the text and replaced the word measure with assess. It is possible that more in-depth training in the form of modules may be a finding of our qualitative interviews with participating pharmacists.***

 In addition to measuring acceptability of the intervention by measuring patient participant engagement would it also be worthwhile to measure health practitioner engagement?

***Response: This is an excellent suggestion. We have been recording this type of data and aim to find out more detailed information from HCP interviews.***

 Aim/question: To understand post-surgical opioid prescribing experiences including opioid use, long-term opioid use, participant pain and quality of life in the 3-month period post-surgery when discharged with an opioid prescription

 It is not clear what the research team means by “Responses during participant interviews.”  Does this mean that it is the intention to interview all study participants or a subset of the study participants?

***Response: The interview is optional and so we expect only a small subset of participants will agree to take part in an interview. We have improved the text around the interview process to make this much clearer.***

 Aim/questions: To ascertain the feasibility of delivering the intervention as per the protocol to inform a future study or adoption of the protocol?

 The protocol suggests that measurements will include a number of process and outcome measures which are indeed important. However, to better understand the feasibility of conducting an intervention the following questions would also need to be addressed:

-
  - Acceptability -from the perspective of the patients and the healthcare providers

***We are capturing this data from participant satisfaction questionnaires and participant interviews and from interviews with key healthcare providers. We have amended the text to make this clearer.***

-
  - what are the required resources (time, personnel, equipment) for implementing the protocol.

***Thi*** **s will be captured in the economic analysis and again we have expanded the text to make this clearer.**

-
  - What are the barriers and facilitators to implementation of the intervention and is it practical.

***This data is being captured as the study progresses via communication with the practices and via drop-in clinical sessions with the clinical lead. Additionally, data will be captured during interviews with HCPs. We plan to interview both, practice sites that engaged and sites that were interested but then did not then proceed to recruit participants.***

-
  - How easily can the intervention be integrated in the existing health care system

***This data is collected under “to ascertain the feasibility of delivering the intervention as per the protocol to inform a future study or adoption of the protocol” I have edited text to say “or adoption of the protocol in the exiting healthcare system”***

-
  - Will the intervention need to be adapted for different primary care/clinical settings

***We collect this data from communication with each of the practices and during site-initiation. I have added this into the objectives.***

-
  - What are the potential challenges in scaling the intervention and what resources are required to sustain the intervention in the long-term?

***The answer to these questions, we hope to determine during the delivery of the study and from the data collected. From a participants’ perspective we will gather important feasibility, randomisation and attrition information. One of the challenges being participants’ recruitment and us being in a good position to understand participants’ response to the study. From a resource perspective, data gathered from day-to-day communication with the sites, the economic analysis and interviews with HCPs should feed into this understanding of potential challenges.***

 While the outcome measures are identified in Table 3 (Summary of study outcomes) it is not clear in the submitted protocol how the data for some of these outcomes will be collected and measured.

 In particular

- Determination of the barriers/enablers that affect the delivery of the intervention from the perspective of the NHS stakeholders
- Determination of the barriers/enablers that affect the delivery of the intervention from the perspective of the
- Determination of the integrity, robustness, and transferability of the protocol for future adoption by the NHS

***We have combined the outcome measures with the aims and objectives in Table 1. We hope that this is now clearer.***
